# Using a digital health platform to implement a multi-component CRC screening intervention within a federally qualified health center: study protocol for a hybrid type I trial

**DOI:** 10.1186/s12913-025-13262-y

**Published:** 2025-08-08

**Authors:** Leah Frerichs, Zhang Zhang, Alexis A. Moore, Isabelle Falk, Xianming Tan, Aliza Randazzo, Alison T. Brenner, Daniel S. Reuland, Ajay Dharod, David P. Miller

**Affiliations:** 1 Department of Health Policy and Management, Gillings School of Global Public Health, University of North Carolina at Chapel Hill, Chapel Hill, NC USA; 2 Lineberger Comprehensive Cancer Center, University of North Carolina at Chapel Hill, Chapel Hill, NC USA; 3 Department of Medicine, Division of General Medicine and Clinical Epidemiology, University of North Carolina School of Medicine, Chapel Hill, NC USA; 4 Department of Biostatistics at Gillings School of Global Public Health, University of North Carolina at Chapel Hill, Chapel Hill, NC USA; 5 Department of Implementation Science, Wake Forest University School of Medicine, Winston-Salem, NC USA; 6 Department of Internal Medicine, Wake Forest University School of Medicine, Winston-Salem, NC USA; 7 Wake Forest Center for Healthcare Innovation (CHI), Wake Forest University School of Medicine, Winston-Salem, NC USA; 8 Wake Forest Center for Artificial Intelligence Research (CAIR), Wake Forest University School of Medicine, Winston-Salem, NC USA

**Keywords:** Digital health technologies, Implementation science, Colorectal cancer screening

## Abstract

**Background:**

Over the past decade, many well-resourced health plans and systems surpassed the 80% colorectal cancer screening rate goal, while lower resource environments such as federally qualified health centers (FQHCs) lag behind. FQHCs in rural areas are especially challenged with limited resources to reach diverse patients who often lack consistent engagement with clinical care. mHealth solutions, like mPATH^®^CRC, can address these challenges by automating tasks and expanding patient outreach. This platform identifies patients due for CRC screening, educates them on the commonly used screening tests, and helps them select their best option. This paper describes protocols for a study that will implement mPATH^®^CRC in FQHCs using a novel outreach strategy that engages patients outside of regular appointments.

**Methods:**

Following a type 1 hybrid effectiveness-implementation design, we will conduct a patient-level randomized controlled trial (RCT) to assess the effectiveness of mPATH^®^-CRC over a three-year period. Embedded within the RCT, we will utilize a convergent, mixed methods design for evaluation of the implementation process. The planned trial sample will include 1000 patients who are at average CRC risk and due for screening. Participants will be randomized 1:1 to receive either usual care or outreach through mPATH^®^-CRC that includes text messages about screening, multi-media education on screening options, and either mailed FIT or assistance to schedule a colonoscopy. The primary outcome is completion of any CRC screening test within six months of randomization. We will also use surveys and interviews with FQHC personnel to explore integration of mPATH^®^-CRC into clinical workflows and sustainability challenges.

**Discussion:**

This trial will generate insights into the effectiveness and implementation of a digital CRC screening intervention in resource-limited FQHCs. Findings will inform strategies for optimizing outreach components and scaling implementation in similar settings.

**Trial registration:**

This trial was registered on June 4, 2024, at ClinicalTrials.gov (identifier NCT06441487).

**Supplementary Information:**

The online version contains supplementary material available at 10.1186/s12913-025-13262-y.

## Background

There are inequities in colorectal cancer (CRC) screening by intersecting characteristics of race, geography, and insurance status. Despite national declines in CRC incidence and mortality due to increased screening, significant disparities remain. Among adults aged 50–75, CRC screening rates are 69% for white individuals compared to 66% for Black, 59% for Hispanic, and 56% for American Indian individuals. Inequities are more pronounced in rural areas where screening rates are 5–6% lower than urban areas across racial groups [1–3]. Furthermore, only 54% of Medicaid and 30% of uninsured individuals are up-to-date with screening compared to 80% or more of those with private insurance or Medicare [4]. Higher screening rates are often driven by well-resourced health plans and systems that have the capacity and resources to implement evidence-based patient and provider outreach and education strategies [5]. Meanwhile, federally qualified health centers (FQHCs) have limited resources to reach patient populations with a disproportionate burden of healthcare needs. It is imperative that research focuses on approaches that reach and resonate with rural, racially diverse patients and the clinics that serve them.

Interventions that combine multiple evidence-based strategies that work in synergistic or complementary ways have been shown to improve CRC screening more than singular strategies [6, 7]. For example, decision aids, especially among low-income Black, Indigenous, and People of Color (BIPOC), may help address issues of mistrust in healthcare, potential low literacy, and other language barriers [8–12]; however, decision aids alone are unlikely to address navigating multi-step screening processes. Thus, decision aids can be more effective when combined with patient navigation [13]. Similarly, mailed FIT (for completion at home and return by mail) has been shown to be effective especially when combined with mailed or phone-based reminders and instructions [14]. However, despite evidence of the effectiveness of these strategies, implementation in settings such as rural FQHCs has been limited. Such multi-component interventions are often too cumbersome and resource intensive for FQHCs to implement due to the limited time and effort capacity of their personnel. Given the resource limitations and inconsistent patient engagement in FQHCs, mPATH^®^ (mobile Patient Technology for Health) CRC offers a scalable solution by automating patient outreach, providing accessible education, and overcoming literacy barriers. By delivering intervention components outside of regular appointments, mPATH^®^ CRC has the potential to mitigate many of the challenges faced by rural clinics and improve CRC screening rates among diverse, underserved populations.

mPATH^®^CRC was designed to “identify patients due for CRC screening, educate them on the commonly used screening tests, and help them select their best option, with the goal of increasing CRC screening rates” [15]. Thus far, mPATH^®^-CRC has been used in primary care clinics wherein patients use an iPad during check-in to view a decision aid about CRC screening options and answer questions about their screening preferences [15]. In a randomized controlled trial conducted in primary care practices affiliated with a large academic health system, an iPad version of mPATH^®^-CRC doubled the proportion of patients who completed CRC screening compared to usual care (30% versus 15%) [16]. However, while this study found mPATH^®^CRC to be effective as a clinical in-reach strategy (i.e., used for patients with appointments), less is known about factors effecting implementation and effectiveness of this type of technological solution with lower-resourced FQHCs that serve patients with relatively lower socioeconomic status. Particularly, delivery during appointments is challenging for FQHCs that have limited staff time and patients with less consistent appointment routines.

This study will implement mPATH^®^CRC with FQHCs using a novel outreach strategy that identifies patients and delivers intervention components to patients outside of regular appointments. This outreach strategy could increase reach and mitigate implementation barriers. The effectiveness will be evaluated using a pragmatic randomized controlled trial designed and anchored in the Process Redesign Framework for understanding both effectiveness and implementation in resource-constrained settings.

## Methods/Design

We will conduct a patient-level, pragmatic randomized controlled trial (RCT) to assess the impact of mPATH^®^-CRC on CRC screening rates over a three-year period. As a pragmatic trial, our goal is to determine if the intervention can improve current practice. Thus, the comparator arm patients will be assigned to usual care. Embedded within the RCT, we will use a convergent, mixed methods design for evaluation of the implementation process over the three-year implementation period. Implementation measures will also be used to understand unintended trial effects. All protocols have been reviewed and approved by the University of North Carolina at Chapel Hill and Wake Forest University institutional review boards, which granted a waiver of informed consent. Multiple data sharing, management, and security agreements were also reviewed and completed amongst UNC, FQHC, mPATH^®^-CRC, and other relevant partners. SPIRIT guidelines were used for reporting [17].

### Setting

Our study will be in rural southeastern North Carolina (NC) and work directly with an FQHC in the area. The FQHC operates eight health clinics in three rural counties, all of which use Epic for their EHR. These counties are the poorest in NC in both health and economic resources [18]. The FQHC is the main safety-net provider for the low-income population and serves between 12 and 15,000 patients a year, over 6,000 of whom are eligible for CRC screening. Nearly all of the FQHC’s patients are at or below 200% of federal poverty guidelines (97%), and half are on Medicaid or Medicare, one-fourth have private insurance, and one-fourth are uninsured [19]. Patients of this FQHC are racially and ethnically diverse, including American Indian (36%), African American (30%), and Hispanic/Latino (25%) patients [19]. In 2023, 72.5% of their patients were overdue for CRC screening [19].

### Participants

#### RCT participants

We will work with the participating FQHC to develop and validate a query of their EHR to identify patients aged 45 to 73 who are average risk for CRC, overdue for CRC screening, and have had at least one clinic visit in the past 12 months. We will exclude patients with a history of CRC or adenomas, total colectomy, family risk of CRC, or diagnosis of inflammatory bowel disease. Overdue for CRC screening is defined as no stool-based (FIT, FOBT) screening within the past one year, no stool DNA test within the past three years, and no colonoscopy within the past ten years.

#### Implementation evaluation participants

We will invite all personnel in the FQHC’s clinics to participate, including primary care providers (physicians, physicians assistants, nurses), clinic staff (clerical and information systems/electronic health record management), and leadership. The only inclusion criteria are that they are employed by the participating clinical partner organization.

### RCT sampling and procedures

**Fig. 1:**
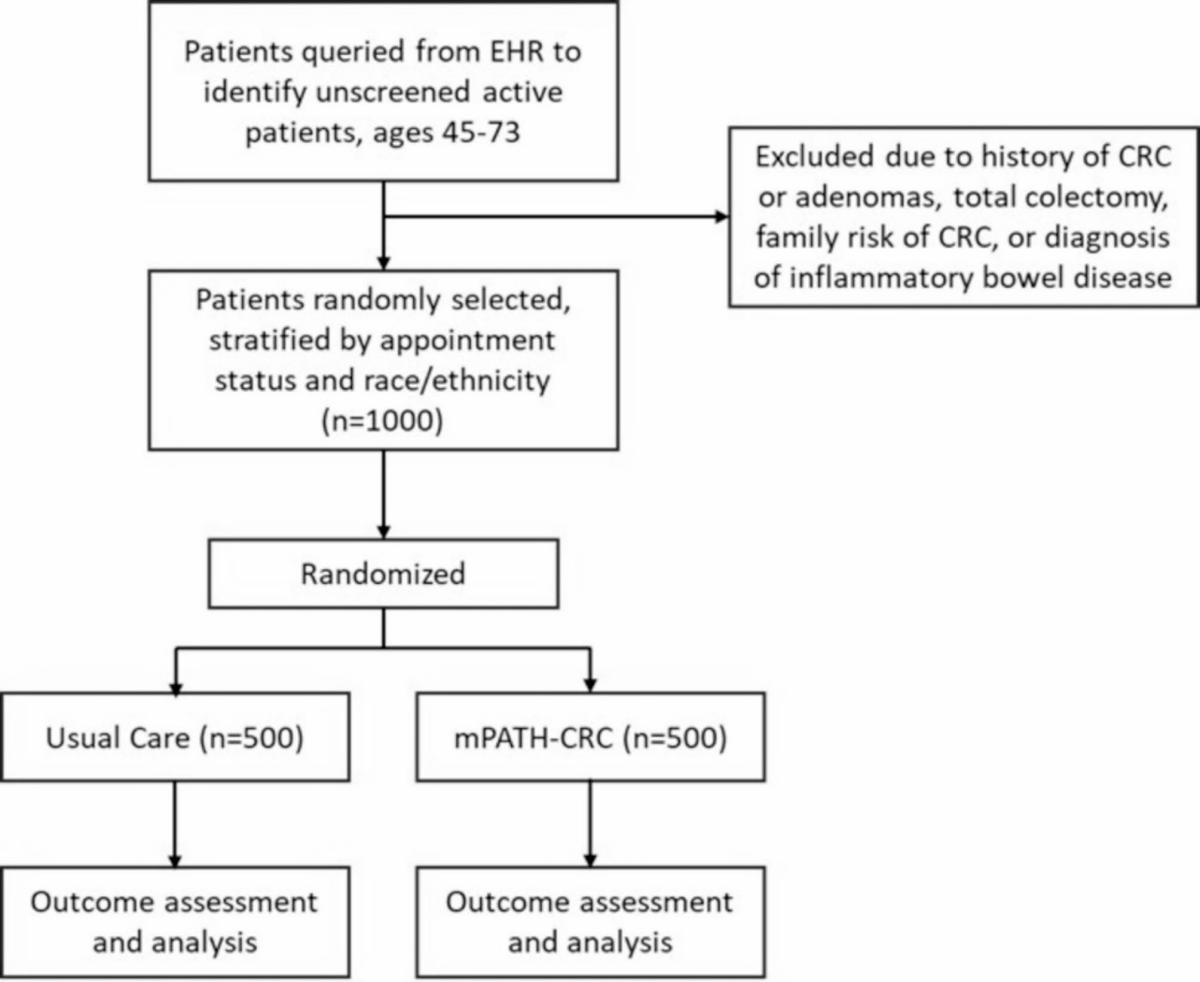
Study flow diagram

Figure 1 provides the study flow. A more detailed study schema is found in the Supplementary Materials. The FQHC will send an EHR query of all eligible (active) patients from their clinic sites approximately once per week to the research team (this list will be updated regularly to include newly added patients and remove previously selected patients or those who become current with screening). A biostatistician on our research team will use a computer-generated random sequence to select patients from the list within strata of appointment status and race/ethnicity: equal patients with an upcoming clinical appointment within approximately 21–45 days and patients without an upcoming clinical appointment (or an appointment greater than 45 days), and within each appointment status strata, equal patients from each of the four race/ethnicity groups represented in the FQHC’s patient population (White, Africa American, Hispanic, and American Indian). Next, we will randomize 1:1 to the mPATH^®^-CRC intervention and usual care within each appointment status and race stratum. We will repeat these sampling procedures until we reach our target sample of 1000 total patients after 8 months. We will follow this cohort for three total years, repeating the same outreach each year dependent on screening modality and completion (i.e., completion of colonoscopy in year 1 would no longer require follow-up, completion of FIT would require annual testing).

Contact data for patients selected for the intervention will be uploaded to the mPATH^®^-CRC platform via a secure file transfer protocol of a flat file to be queued for intervention delivery. It is not possible to blind patients to this type of intervention; however, the materials will be branded with their FQHC’s information, and patients will not be informed that a usual care arm exists. Similarly, usual care participants will not be informed of the study. Outcome assessors will be blinded to study arm.

### Implementation evaluation sampling, procedures

Intervention patients will be surveyed post-randomization using text messages containing links to an online survey and inviting them to rate their satisfaction with the digital outreach and services for CRC screening. All respondents will receive a $10 gift card for each survey completed.

**Fig. 2:**
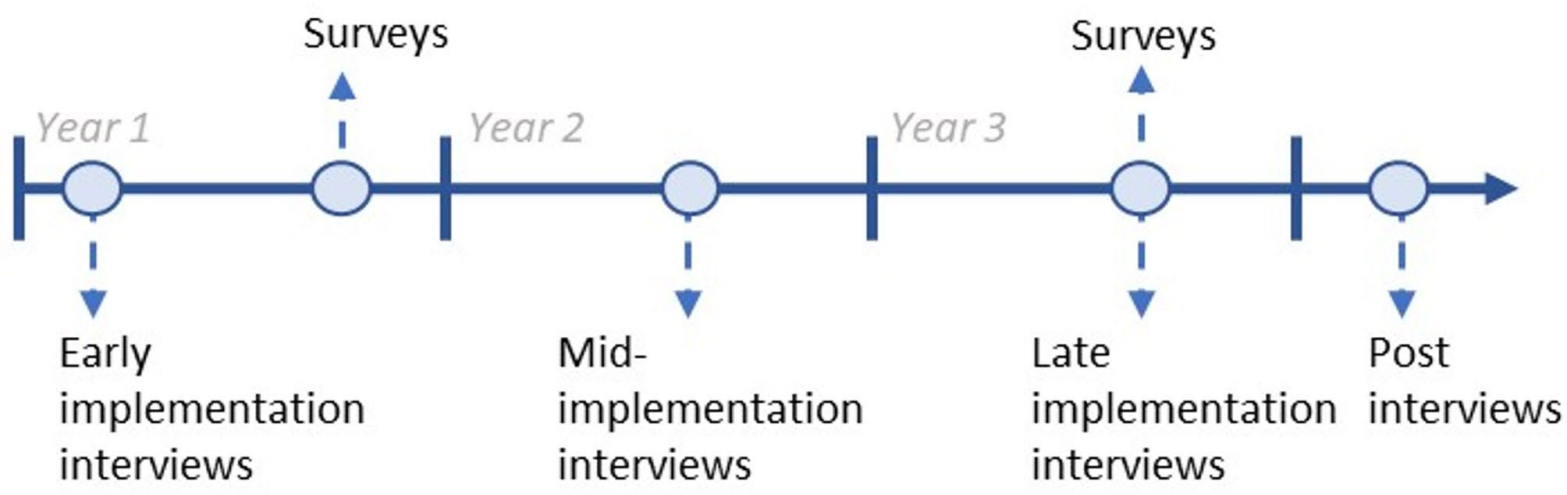
Provider and staff data collection timeline for implementation evaluation

As indicated in Fig. 2, we will conduct semi-structured interviews with providers and staff (*N* = 16 or until theme saturation is reached) and surveys with all clinic providers and staff (*n* = 210) at specified time-points. We will use purposive sampling to recruit interviewees who have the most involvement in the intervention (e.g., IT staff who assist with EHR queries, leadership who make decisions regarding clinic-level programs and practices). Administrative leaders will be asked to endorse and invite participation. Trained research staff will be interviewers for semi-structured interviews. All participants will be offered a $10 and $50 gift card for each completed survey and interview, respectively.

### Intervention and comparisons

#### Usual care arm

Patients assigned to usual care will receive the normal care and services used at the FQHC. For this clinic, this primarily involves clinical in-reach by providers during scheduled appointments to assess and recommend a screening modality including FOBT/FIT or referral for colonoscopy.

#### Intervention arm

As shown in Fig. 1, patients selected for the intervention will be sent a text message that indicates they may qualify for a CRC screening and provides a link for further information, which directs them to the mPATH^®^-CRC interface. Patients without cell phone numbers will be mailed an invitation with the same information and a tiny URL and personal code to access the interface, although we anticipate this will be < 5% of participants. Within the mPATH^®^-CRC interface, patients will first answer a series of questions to confirm CRC screening eligibility and assess for risk factors that would change screening recommendations. If eligibility for routine CRC screening is confirmed, they will view a previously validated decision aid about CRC screening that provides information about their testing options, i.e., FIT or colonoscopy. After the video, patients are given the option to “self-order” either of those tests.

The FQHC will have its own secure mPATH^®^-CRC dashboard that displays patient requests for screening tests. The mPATH^®^-CRC system automatically emails designated staff a secure link to the dashboard when new patient screening requests occur. Notices of patients who select colonoscopy will be sent to the FQHC’s referral coordinators for further scheduling and communication. To integrate with the FQHC’s FIT processing and communication, patients who select FIT will be mailed a FIT kit using the FQHC’s usual FIT and lab vendors. Any patients who fail to engage with mPATH^®^-CRC interface within approximately 2 weeks of the initial text or mailed invitation will be mailed a FIT kit by default. The research team will have access to the mPATH^®^-CRC dashboards for implementation evaluation and will assist with prompting patient follow-up care and assistance, as appropriate.

All interface materials and communication will be offered in either English or Spanish, dependent on the patient’s language preference, including the decision aid video. Participants will also have the option to view a decision aid developed and culturally adapted in partnership with tribal communities in the state [20].

### Measures

#### RCT primary outcomes

Since patients can decide between FIT or colonoscopy, the primary outcome will be CRC screening completion defined as meeting one of the following mutually exclusive pathways: (1) colonoscopy completion (regardless of indication), (2) at least one FIT test with normal FIT result, or (3) diagnostic colonoscopy following an abnormal FIT result. This measure is standard and comparable with prior CRC screening intervention studies [13, 21]. We will measure this outcome 6 months after randomization for all participants (including usual care). Outcomes will be based on encounter level data queried from the EHR, which mitigates issues of participant retention and provides pragmatic results.

#### RCT secondary outcomes

We will also conduct a secondary analysis using years of CRC screening coverage over the three-year study period as the outcome. FIT must be repeated annually, and colonoscopy typically only needs to be repeated once every 10 years. Thus, patients will be assigned one year per annual FIT completed (e.g., FIT in years 1, 2, and 3 would be assigned 3 years; FIT in year 1 and 3 would be assigned 2 years) and up to 3 years for colonoscopy depending on the year received (e.g., colonoscopy in year 1 would be assigned 3 years, colonoscopy in year 3 would be assigned 1 year). Patients with a positive FIT result must complete a colonoscopy to count as covered. We will also collect patient-level data from the EHR record on appointment status, race, ethnicity, age, gender, insurance status, co-morbidities, and prior CRC screening to use as covariates or stratified analyses as appropriate.

#### Patient process and implementation measures

We will also invite patients randomized to the intervention to complete an online survey that will assess satisfaction and acceptability of digital interventions using constructs from the Technology Acceptance Model (e.g., ease of use, perceived helpfulness, and perceived usefulness) [22–25], decisional satisfaction [26, 27], and an assessment of culturally and linguistically appropriate care [28]. Details on the measures are provided in Table 1.

**Table 1:** Implementation survey measures for clinical providers and staff and patients

Provider and staff survey	Description	Source
Acceptability	Eight items with a 5-point ordinal rating scales that measure seven constructs from the theoretical framework of acceptability including affective attitude, burden, ethicality, intervention coherence, opportunity costs, perceived effectiveness, and self-efficacy.	Theoretical Framework of Acceptability questionnaire [25]
Sustainability	Seven items that measure capacity for program sustainability, including within domains of engaged staff and leadership, engaged stakeholders, organizational readiness, workflow integration, implementation and training, monitoring and evaluation, outcomes and effectiveness	Adapted from Clinical Sustainability Assessment Tool [31]
Cultural Sensitivity	14 items with 5-point ordinal rating scale that ranged from “not at all” to “excellent” that provide a self-evaluation of knowledge (6 items), skills (6 items), and attitudes (4 items) relevant to cultural sensitivity.	Adapted from Culhane-Pera et al.’s Multicultural Assessment Questionnaire [32]

Patient survey	Description	Source
Acceptability of digital interventions	Twelve items that assess ease of use (e.g., “It was easy to use mPATH-Cloud”), trust (e.g., “I trust the information provided by mPATH-Cloud about CRC”, and overall likeability (e.g., “I would recommend mPATH-Cloud to a friend or family member”) of the digital intervention on 5-point rating scales (1 strongly disagree; 5 strongly agree)	Adapted from acceptability and ease of use surveys [22–24]
Decisional satisfaction	Six items (e.g., “The decision I made was the best decision possible for me personally”) that assess patient satisfaction with a health care decision, rated on a 5-point ordinal rating scale (1 strongly disagree; 5 strongly agree)	Satisfaction with Decision scale [26, 27]
Cultural sensitivity	Six items (e.g., “my doctor understands my background and values”) that assess perceived provider (3 items) and health system (3 item) cultural sensitivity	Bias and Cultural Competence measures [28]

#### Clinic provider and staff implementation measures

Table 2 provides an overview of the measures for provider interviews and Table 1 provides an overview of provider/staff survey measures. Our evaluation of implementation outcomes is guided by the process redesign framework (PRF) [29]. We will include measures within each domain of the PRF but will focus on those most relevant to this project. Specifically, emphasis will be placed on specific domains at different stages of the study. Early implementation interviews will focus more heavily on general processes for CRC screening and the integration and redesign of those processes for effective and efficient implementation of mPATH^®^-CRC. For provider surveys, we will also use validated surveys including Weiner et al.’s Acceptability of Intervention Measure, Feasibility of Implementation Measure, and Intervention Appropriateness Measure [30], Clinical Sustainability Assessment Tool [31], and Culhane-Pera et al.’s Multicultural Assessment Questionnaire [32].

**Table 2:** Implementation interview constructs and measures

Constructs	Description or Guiding Questions
**Clinic Staff and Provider Early Implementation Interviews**	
***Intervention Characteristics***	
Workflows	What are the current and needed changes to processes and tasks required of interconnected stakeholders (providers, IT staff, FIT processing lab) to implement mPATH^®^-CRC
Tasks/Process Standardization	In the process design for implementation, which tasks and processes can be standardized and what are the protocols for standardization across interconnected stakeholders
***Outer Setting***	
Technological Changes	Are there changes in technology (e.g., acceptability, cost, etc.) that may impact mPATH^®^-CRC?
***Inner Setting***	
HIT/IT systems	What is the current technology infrastructure (e.g., EHR) and what is required to support integration of mPATH^®^-CRC for managing patient care, data, and communication?
Staff Resources	What is the staff time required to assist with implementation tasks such as querying patients due for screening, quality assurance of data, follow-up of abnormal FIT, etc.?
**Clinic Staff and Provider mid-term implementation interviews**	
***Implementer Characteristics***	
Skills/Competencies	What is the expertise, skills, and competencies of implementation stakeholders relevant to mPATH^®^-CRC implementation? To what extent do providers at mPATH^®^-CRC implementation sites exhibit cultural humility and linguistic sensitivity to their patients?
Roles	What are the individual and team roles/responsibilities for implementation and to what extent are there multiple/shared roles for implementation of mPATH^®^-CRC?
***Process***	
Acquiring/allocating resources	What are the major resources dedicated to implementing mPATH^®^-CRC (staff time, space, equipment)? To what extent are those resources adequate or inadequate?
Process ownership	Who is making mPATH^®^-CRC implementation decisions? How often are decisions made and how involved are decision-making processes? Is the approach top-down, bottom-up, a blend?
Decision making	Who has a major role in implementing mPATH^®^-CRC? What are their role(s) and how are they varied/different? In what ways, if any, has there been authority, accountability for these roles?
**Clinic Staff and Provider late implementation interviews**	
***Implementation***	
Penetration	To what extent have processes and tasks for implementation of mPATH^®^-CRC been integrated into the clinic’s workflows and other subsystems such as the EHR?
Replicability	To what extent is mPATH^®^-CRC perceived as replicable in other settings and clinics?
Evolvability	Does mPATH^®^-CRC require continued adaptations and refinements for sustainability? If any, what are these types of adaptations and changes?
Sustainability	To what extent are the clinic leadership, staff, and providers interested in sustaining the use of mPATH^®^-CRC? What do you think would be needed to sustain mPATH^®^-CRC?

### Analyses

#### RCT primary outcome analyses

For the RCT design, our main statistical test for the primary outcome (CRC screening completion) will be a Mantel-Haenszel chi squared test, adjusted for stratification factors (appointment status, race/ethnicity), following the intention to treat principle. If there are differences between the arms across baseline variables known to be associated with the outcome, we will use multiple logistic regression, adjusting for the variables. Specifically, due to the potential for differential response, sex will be included in all analyses as a biologic variable. One-sided tests will be used because we expect active intervention will lead to more favorable results compared to usual care. Under the intention to treat principle, our primary analysis will be a crude comparison of proportions of completed CRC screening at six months between the mPATH^®^-CRC and usual care arm using a one-sided Mantel-Haenszel chi-squared test, controlling for stratification factors (appointment status and race/ethnicity), conducted at the 0.05 significance level. As a minimal risk study using only strategies known to improve evidence-based screening, we will not convene a formal data monitoring committee.

#### RCT secondary analyses

We will compare CRC screening coverage between the two groups using the Wilcoxon rank sum test. Additionally, we will calculate descriptive summaries (mean, standard deviation, median, and range) for the entire sample and by group.

#### RCT sample size and power

Based on our prior studies (33–37), we anticipate CRC screening rates will be between 5 and 12% in usual care and 10–35% in the mPATH^®^-CRC arm, with at least a 5–7% absolute difference between arms, conservatively. With *n* = 500 in each arm, we will have more than 80% power to detect such differences (5–7%) between the arms at 1-sided alpha level 0.05.

#### Implementation evaluation analyses

For the hybrid evaluation of implementation process, we will use deductive analysis to explore contextual domain themes. All interviews will be audiotaped, transcribed, and checked for accuracy. A codebook with definitions will be developed using an iterative process and reviewed for face validity by experts independent of the research team. Two research staff will review and code each transcript independently using the coding framework to assess themes within each of the domains of interest (intervention and implementer characteristics, outer setting, inner setting). Coding discrepancies will be reconciled by team consensus. We will summarize survey data using descriptive statistics, including frequencies for overall scores for acceptability and appropriateness. Based on our prior experiences, we anticipate survey response rates of 50–60%, which will allow us to estimate the proportion who agree with any one survey item to within +/- 7% for these descriptive and exploratory analyses.

## Discussion

We described a protocol for a type 1 hybrid effectiveness-implementation trial to assess the impact of a digital outreach platform, mPATH^®^-CRC, on CRC screening completion among patients and process, acceptability, and sustainability measures among clinical providers and staff. Although prior studies have shown the effectiveness of the component strategies of our proposed research (text/electronic reminders, decision aids, mailed FIT) [7, 38, 39], the planned study will examine their effectiveness as a novel bundled package using a digital platform that aims to improve implementation support for low-resource settings that serve racially and ethnically diverse populations. Our study will fill critical research gaps about patient level interventions combined with clinic level implementation strategies and has potential to improve CRC screening rates among populations that continue to lag behind national increases.

As with any study, we anticipate encountering challenges common to pragmatic research with patients with limited resources. Although prior studies have shown the effectiveness of text-based reminders for CRC screening [38, 40], our study uniquely uses text messaging to engage patients in education and “self-ordering” a test. We have evidence that the digital divide is shrinking [41] and many of the patients in our study will have appropriate technology and willingness to engage, but this may be a challenge especially as communication overload and messaging fatigue is becoming more of a concern [42]. As such, the intervention will be branded by the FQHC (to improve trust) and we planned alternative strategies to reach patients without cell phones (via mail) or who fail to respond (default mailed FIT). Our study will carefully collect data on characteristics of patients to improve understanding of who is most likely to engage with the mPATH^®^-CRC platform as well as the modalities of screening preferred and completed. This data will be useful for future adaptations to the multi-component strategies and may present opportunities for more personalized messaging.

We also anticipate challenges at the clinic level. As with many low-resource settings, we expect staff turnover [43, 44] and the willingness and acceptance of clinical staff to adopt innovations in technology [45–47] to be specific challenges. Our implementation evaluation will be important to better understand these challenges in greater detail to inform future implementation strategies.

In summary, the proposed study has considerable potential to improve knowledge about effectiveness and implementation of multi-component CRC screening interventions in FQHCs and similar settings. We anticipate using the knowledge gained to inform future iterations of the multi-component strategies. For example, potentially using the technology-based platform to improve tailoring of messaging and outreach based on key demographics or screening indicators. Furthermore, we anticipate generating more robust implementation strategies that can be tested in type 2 or 3 hybrid effectiveness-implementation trials with a greater emphasis on implementation. Finally, we intend to disseminate these findings both locally (to help the FQHC adapt and sustain the intervention) as well as nationally through publications in peer-reviewed journals and scientific meetings to improve use of our lessons learned and improve CRC screening among diverse populations in a variety of contexts.

## Contributions to the literature

- This paper describes a digital health platform, mPATH-CRC^®^, that serves as both an implementation and a patient-centered strategy to increase colorectal cancer (CRC) screening by automaking tasks for clinics while expanding culturally-sensitive patient outreach and education.
- In partnership with a rural federally qualified health center, this study will evaluate the impact of mPATH-CRC^®^ on CRC screening outcomes, while collecting quantitative and qualitative data on the integration of mPATH^®^-CRC into clinical workflows and sustainability challenges.
- The study results will provide new knowledge about how to use digital health strategies to optimize patient outreach and education while providing a scalable implementation strategy.

## Supplementary Information

Below is the link to the electronic supplementary material.

Supplementary Material 1

Supplementary Material 2

Supplementary Material 3

## Data Availability

No datasets were generated or analysed during the current study.
