# Supplementary figures and images for: Using a digital health platform to implement a multi-component CRC screening intervention within a federally qualified health center: study protocol for a hybrid type I trial
Source: BMC Health Serv Res. 2025 Aug 8;25:1047. doi: 10.1186/s12913-025-13262-y (PMC12335168; doi:10.1186/s12913-025-13262-y)

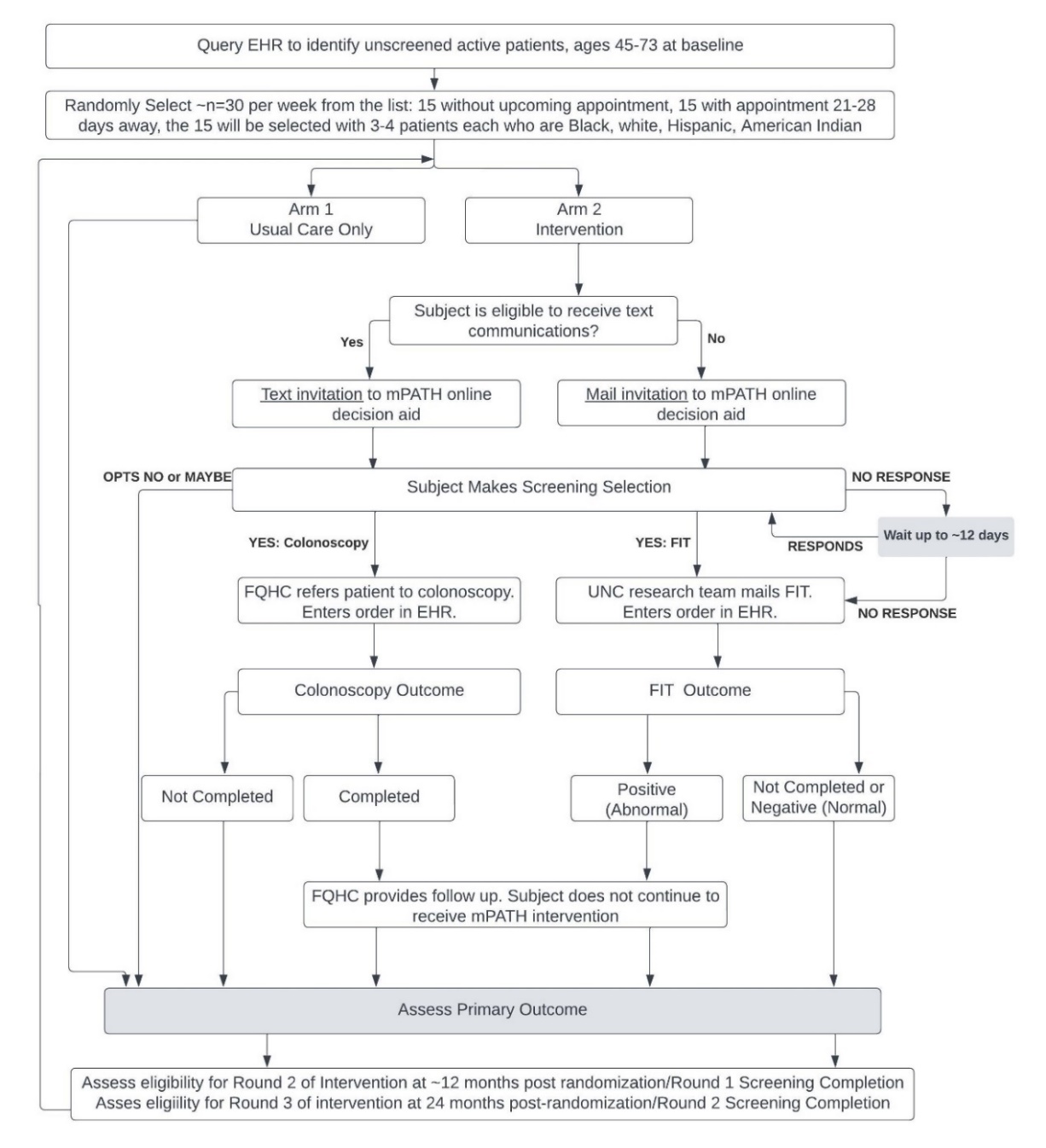


**Supplementary Figure. Study Schema**

Supplement: Supplementary file 1 — Supplementary Material 1 [file 12913_2025_13262_MOESM1_ESM.docx]
